# Clinical Experience With Intramuscular Clozapine

**DOI:** 10.7759/cureus.18267

**Published:** 2021-09-25

**Authors:** Benedikt Munzar, Boris Nemets

**Affiliations:** 1 Department of Psychiatry, Ben-Gurion University of the Negev, Beer Sheva, ISR

**Keywords:** schizophrenia, compulsory treatment, parenteral injection, intramuscular, clozapine

## Abstract

Clozapine is the most effective antipsychotic for patients with treatment-refractory schizophrenia, but many refuse to accept oral clozapine therapy. Intramuscular (IM) clozapine represents a convenient alternative for their treatment. The aim of this review is to summarize studies investigating IM clozapine administration. When initially developed, clozapine was also provided as an IM formulation, but the manufacturer later discontinued its production. Recently, IM clozapine became again available as an unlicensed product distributed by the Dutch company Apotheek A15. The use of IM clozapine has been reported in older studies on clozapine’s adverse effects. It has also been described in detail in 5 more recent and generally smaller (n = 7 - 59) retrospective studies in patients refusing to take oral clozapine. In addition, its administration has been noted in 5 case reports. IM clozapine has been used at approximately ½ of the dose of oral clozapine due to pharmacokinetic considerations. It has been used in doses of up to 500 mg per day and for up to 99 days of treatment. The majority of patients (between 60 and 100%) were successfully transitioned to oral clozapine within a few days of IM treatment, and improvement in their condition was sustained during the long-term follow-up. Side effects of IM clozapine were similar to those of oral clozapine, but its sedative and cardiovascular effects (hypotension and tachycardia) had faster onset following IM administration. After long-term use, clozapine injections lead to local swelling and to the formation of painful nodules in some patients. In summary, IM clozapine may facilitate successful transition to oral clozapine in most patients, and it definitely represents a valuable tool for addressing refusal of oral clozapine in patients with treatment-refractory schizophrenia. More studies, especially focused on its safety, are, however, needed to better understand the limitations of this novel treatment approach.

## Introduction and background

Clozapine is the most effective antipsychotic for patients with treatment-refractory schizophrenia, providing clear benefits in about 60% of subjects [1-3]. However, many treatment-refractory patients are refusing to accept oral clozapine therapy, with the percentage of noncompliance estimated at about 20% [2, 4] and are thus deprived of this life-changing treatment. In addition to those refusing oral treatment, there are subgroups of patients who are unable to accept oral formulations of clozapine due to swallowing difficulties or other GI problems [5-6], cannot receive oral clozapine due to perioperative status [7], are unable to benefit from oral clozapine due to catatonia precipitated by clozapine discontinuation [8] or caused by other factors responsive to clozapine treatment [9]. There is thus a significant unmet medical need for alternative non-oral delivery routes for clozapine administration.

Clozapine may be mixed with food offered to non-consenting subjects without their knowledge [10]. This option is, however, very unreliable and is considered unethical by most psychiatrists [11]. At the moment, the only readily available reliable way for delivering clozapine to patients refusing to take it is via nasogastric administration [6, 11-13]. That option requires restraints and is traumatizing to both the patient and attending staff and is thus reserved only for the most complicated cases where no other alternative exists [14]. Alternative delivery routes, such as nasal delivery [15] and transdermal delivery [16-17], are in development but are not yet clinically available. Clozapine has also been delivered via intravenous infusion but only under a research setting [18].

An easy alternative route to oral clozapine delivery would be its intramuscular administration. When clozapine was initially developed, it was also available as an intramuscular formulation and was used in most European countries as well as in Israel but was never registered in the United States [2]. The manufacturer discontinued that formulation due to commercial reasons in 2005, and its utility has become quite limited due to the need to use compounding pharmacies [19-20]. However, intramuscular clozapine has gained popularity over the past few years due to the distribution of unlicensed product made by a Dutch Apotheek A15. Its use has been reported in several recent studies and case reports from the United Kingdom [6, 21-24].

In this review, we summarize all available reports on intramuscular clozapine administration and address reported efficacy as well as potential side effects and risks associated with this clozapine formulation.

## Review

Methods

We searched for articles indexed in the PubMed, Medline, Google Scholar, and ScienceDirect databases up to August 15, 2021, utilizing regular search keywords such as "clozapine," "intramuscular," "parenteral," "injection," "non-oral," "enforced," and "compulsory," which were used in various combinations. Articles generated were further checked for the relevance to the topic of the study, and cited references were investigated. In addition, meeting abstracts were reviewed. Finally, selected authors of identified reports were queried for availability of any additional data. After detailing information on intramuscular clozapine formulation, each identified study is described in depth below.

Intramuscular clozapine formulations

The initial intramuscular formulation of clozapine was marketed by Sandoz and later by Novartis but Novartis stopped commercially producing intramuscular clozapine in 2005 due to economic factors [19-20]. It was subsequently made by various compounding hospital pharmacies, but its utility remained limited [19]. Presently, clozapine ampules are offered by Apotheek A15 company (formerly known as Brocacef) in the Netherlands (for package insert, see [25]). However, the Dutch product is not a licensed product and is distributed only under special permissions from regulatory authorities in individual countries. For example, the British NHS Foundation Trust established complex protocols for administering that clozapine formulation under rigorously controlled conditions [26]. It is costly, with one ampule being sold for about $160. Clozapine ampules are also available from El Saad Pharma in Syria (for package insert, see [27]).

All formulations of intramuscular clozapine contain clozapine at the 25 mg/mL concentration. Apotheek A15 clozapine formulation is distributed in 5 mL ampules. There is thus 125 mg of clozapine in each ampule. Per the recommendation of its producers, it should be administered into a gluteal muscle, and if more than 100 mg is needed, the dose should be divided into two injection sites. 

As oral bioavailability of clozapine is approximately 30 - 50% [18, 28] it is recommended to use ½ dose of oral clozapine if intramuscular substitution or initial titration are done [26]. However, it has to be noted that there is no published study on pharmacokinetics of intramuscular clozapine, and clozapine levels after its intramuscular administration are likely transiently much higher than after its oral intake, and that may have consequences for its safety.

Summary of studies

All available studies mentioning intramuscular clozapine, including case reports and surveys, are summarized in chronological order in Table 1.

**Table 1:** Studies mentioning intramuscular clozapine listed in a chronological order

Reference (First author, publication year, reference number)	Study description	Study outcomes related to IM clozapine administration	Number of subjects getting IM clozapine	IM clozapine daily dose range/duration
Lokshin 1999 [4]	Retrospective chart review on patients receiving IM clozapine	IM clozapine led to clinical improvement and to acceptance of oral medication	59	50 – 400 mg/ 3 – 8 days
Gee 2021 [6]	Case report on 2 medically compromised patients	IM clozapine effective in controlling psychosis	2	Up to 150 mg/more than 7 days
Pereira 1999a [11]	Survey of psychiatrists on enforcing clozapine administration	Six out of 39 psychiatrists reported using IM clozapine in patients refusing to accept oral	not reported	not reported
Holmes 2019 [21]	Retrospective chart review on patients getting IM clozapine	IM clozapine was well tolerated and allowed initiation or continuation of oral clozapine	7	not reported/ 1 – 4 days
Casetta 2020 [22]	Retrospective chart review on IM clozapine	Sixteen out of 19 patients successfully transitioned to oral clozapine, and most remained compliant during long-term follow-up	19	6.25 – 200 mg/ 1 – 47 days
Henry 2020 [23]	Retrospective chart review on IM clozapine	Six out of 8 patients receiving repeated IM clozapine injections successfully transitioned to oral clozapine	8	Up to 250 mg/ 1 – 99 days
Whiskey 2020 [24]	Case report on a patient with schizoaffective disorder and heart failure	IM/oral clozapine titration led to stabilization of the mental state	1	not reported
Ackenheil 1989 [29]	Pharmacokinetic and biochemical investigation in schizophrenic patients	Changes in biochemical parameters similar to oral administration	13	100 mg/1 day
Grohman 1989 [30]	Post-marketing drug surveyance program in 959 patients on both oral and IM clozapine	Death in 1 patient after IM clozapine (given together with haloperidol)	not reported	100 mg/1 day
Marinkovic 1994 [31]	Side effects in 100 hospitalized patients (on both oral and IM clozapine)	Incidence of side effects reported (most frequent side effect was tachycardia, and it often occurred immediately after IM dose)	not reported	50 mg and higher/not reported
Gaszner 2002 [32]	Study on agranulocytosis incidence in 750 patients on clozapine (both oral and IM administration)	Granulocytopenia developed in 1 patient who was initially on IM clozapine but 4 years after continued oral treatment	not reported	not reported/ ”several days”
Barak 1999 [33]	Report on clinical experience with clozapine in elderly (both oral and IM)	IM clozapine terminated in 1 patient due to elevated liver enzymes	1	12.5 – 37.5 mg/ 3 weeks
Schulte 2007 [34]	Retrospective chart review of patients on compulsory clozapine treatment	Clinical global impression – improvement noticed in patients on IM clozapine	10	12.5 – 500 mg/ 1 to 90 days
Heim 1994 [35]	Case report on 1 patient on IM clozapine	Dyskinesia developed after IM injection but good clinical improvement noticed	1	100 – 150 mg/ 8 days
McLean 2001 [36]	Case report on a treatment-resistant patient with schizoaffective disorder	IM clozapine led to a successful transition to oral clozapine	1	12.5 – 250 mg/ 30 days
Kasinathan 2007 [19]	Case report on 2 patients with treatment-resistant schizophrenia	IM clozapine led to the rapid acceptance of oral clozapine and sustained improvement	2	12.5 – 100 mg/1 to 7 days
Andersen 2014 [37]	Registry research on the involuntary treatment of schizophrenic patients in Denmark	Two patients were treated with IM clozapine	2	Up to 420 mg/ not reported

 As above, there is no published study available evaluating the pharmacokinetics of intramuscular clozapine. However, one experimental study on selected biochemical effects of clozapine in 32 hospitalized schizophrenics reported also outcomes in 13 patients who received 100 mg of clozapine intramuscularly [29]. No apparent differences between oral and intramuscular administration of clozapine in levels of catecholamines and their metabolites as well as in hormonal secretion were noted. Side effects of treatment included orthostatic dysregulation and sedation, but no differences between various routes of administration were reported.

In a large safety surveyance study on adverse effects of clozapine in 959 patients exposed to any formulations of clozapine in Germany, common side effects were reported in 76% of patients [30]. However, only 3.9% of patients experienced side effects that were considered severe. Sudden death was noted in a 31 years old female patient. She had been on continuous outpatient treatment with oral clozapine 200 mg daily for 2 years. She became psychotic and oral haloperidol 20 mg per day has been added. She had been subsequently admitted to the inpatient psychiatric unit. The day after her admission, instead of oral clozapine, she received intramuscular clozapine at 100 mg together with haloperidol 10 mg (per report PO). Four hours later, she was found cyanotic and pulseless. She had been resuscitated but remained comatose and died 4 days later. Of note, she had a history of surgery for interatrial septal defects 13 years prior to the event. This fatal adverse event was labeled as “possibly” related to a combination of clozapine and haloperidol. Per the authors, “whether the IM administration of clozapine - unique in this patient on the day of the fatal complication - was of particular relevance must remain open to speculation.”

Another study evaluating adverse effects of clozapine in 100 hospitalized patients in Serbia also reported on patients getting clozapine intramuscularly though the number of subjects exposed to that clozapine formulation was not specified [31]. The most common side effects were tachycardia, hypotension, and sedation. Of note, the intensity of those side effects was related to the route of administration as they were commonly observed immediately after intramuscular application of 50 mg or more of clozapine, whereas higher oral doses were required to produce similar effects.

Another large study from Hungary summarized the incidence of agranulocytosis in 750 patients on both oral and intramuscular clozapine [32]. Per the report, “clozapine was administered both parenterally and orally (mainly as tablets). In a proportion of patients, treatment was initiated by administering clozapine twice daily by intramuscular injection and switching the patient over to oral dosage several days later.” Out of 750 patients analyzed in the study, only 2 developed agranulocytosis and 7 granulocytopenia. One patient who developed granulocytopenia was initially getting clozapine intramuscularly, but granulocytopenia developed 4 years later and was thus clearly unrelated to the initial administration route. 

In a study on the use of clozapine in the elderly, one female patient in Israel was treated with intramuscular clozapine due to noncompliance and aggressive behavior [33]. Clozapine treatment in that patient had to be terminated as after 3 weeks of intramuscular clozapine, her liver enzymes were significantly elevated.

In a large study from Israel, intramuscular clozapine was given to 59 psychotic patients [4]. In that study, all the patients were on oral clozapine in the past but then started to refuse to take it, and the intramuscular formulation was initiated once they developed an exacerbation of psychotic symptoms. Intramuscular clozapine resulted in quick sedation in that study and in rapid improvement in behavior. Sixteen patients required only 3 days of parenteral clozapine before restarting the oral. Forty-two patients received clozapine injections for 4 to 7 days before transitioning to an oral formulation. Only one patient required intramuscular administration for 8 days.

In a retrospective study on compulsory treatment with clozapine in Netherland [34], 17 patients refusing oral clozapine were prescribed intramuscular injections, but 7 of them accepted oral formulation when faced with an injectable alternative. However, 10 patients were given at least one injection. Four patients received injections for 4 days or less, 3 patients for 7 to 11 days, and 3 patients for 1 to 3 months. Six out of 10 patients were successfully transitioned to oral clozapine. Intramuscular treatment had to be stopped in 4 patients. One patient continued to refuse oral medication despite receiving intramuscular injections for 90 days. One patient developed leucopenia, and clozapine treatment thus had to be stopped. Injections had to be stopped in another patient who developed swelling at the injection site. Two patients recovered to the point that they convinced appropriate authorities that they did not meet the criteria for involuntary injections anymore.

In a study by Holmes [21], 7 patients were treated with intramuscular clozapine - 5 were exposed to clozapine previously, whereas 2 were clozapine naïve before initiation of the treatment. All of them were successfully transitioned to oral clozapine after no more than 4 intramuscular injections.

In a case series by Henry and colleagues [23], 11 patients prescribed intramuscular clozapine are presented. Out of those, 3 accepted oral clozapine prior to getting the first injection, but the remaining 8 received between 1 and 99 doses per patient. Six out of those 8 patients were successfully transitioned to oral clozapine.

In a retrospective study by Casetta and colleagues [22], 39 patients were prescribed intramuscular clozapine, but most of them accepted oral formulation when an injection alternative was offered. However, 19 patients received intramuscular clozapine, and out of them, 16 successfully transitioned to oral formulation within 1-47 days. Intramuscular clozapine had to be stopped only in 1 patient due to continued refusal, while in the remaining 2 patients, clozapine was stopped due to emergent side effects.

In a case report from Germany, a 51-year-old female was started on intramuscular clozapine at 100 mg per day [35]. Following the first injection, she developed trismus in the half of her face and additional involuntary movements which were interpreted as dyskinesia and resolved after biperiden injection. Intramuscular clozapine treatments were nevertheless continued in that patient for 7 more days, and the dose has been increased to 150 mg without observation of any additional side effects. A good antipsychotic effect was noted, and she was successfully transitioned to oral clozapine.

In an Australian case report by McLean and Juckes [36] clozapine was administered intramuscularly to a 64-year-old female with treatment-resistant schizoaffective disorder, and it has been titrated from 12.5 mg per day up to 250 mg per day. After 30 days of that treatment, the patient was successfully transitioned to oral clozapine.

In another case report from Australia, 2 patients were treated with intramuscular clozapine [19]. Both rapidly accepted oral clozapine - the first one after just 1 injection and the second after 7 days of treatment. 

In a case report by Whiskey and colleagues [24], clozapine was successfully re-initiated using oral/intramuscular titration regimen in a 51-year-old man with treatment-resistant schizoaffective disorder and heart failure.

In a case report by Gee and colleagues [6], intramuscular clozapine was successfully given to two medically compromised psychotic patients. One patient was a 50-year-old female suffering from liver carcinoma who started to refuse her oral clozapine and deteriorated and became aggressive. Clozapine was successfully re-titrated through intramuscular injections (up to 150 mg per day), and she then became again amendable to taking oral clozapine, and her mental status stabilized to the point that she could spend her final weeks together with her family. Another patient was a 47-year-old schizophrenic who jumped from the third floor and suffered significant injury. As a result of spinal fractures, he also exhibited swallowing difficulties and received treatments, including clozapine, via nasogastric tube. He later removed the tube and refused to allow re-insertion. For that reason, he was successfully switched from oral clozapine 300 mg per day to 150 mg of intramuscular clozapine, and his mental status rapidly stabilized. Once he regained swallowing abilities was transitioned back to oral clozapine.

In a survey of 39 British psychiatrists, 6 reported enforcing intramuscular medication each time oral clozapine was refused [11]. In a register-based study from Denmark, it was reported that 2 patients with schizophrenia were receiving intramuscular clozapine involuntarily during a studied period [37].

Efficacy outcomes

Most studies reporting on the efficacy of intramuscular clozapine focused on successful transition to the oral product as the primary outcome. Some studies also followed patients long-term after initial intramuscular treatment and reported on their long-term compliance with oral clozapine.

Successful transition to oral clozapine has been achieved in all published case reports [6, 19, 24, 35-36]. In a report by Kasinathan and Mastroianni [19], 2 patients who successfully transitioned to oral clozapine were followed for 12 months, and improvements induced by initially enforced clozapine therapy were sustained.

In published retrospective studies, the majority of subjects transitioned from intramuscular to oral clozapine within a few days. In the largest report by Lokshin and colleagues [4] in 59 patients, almost all the patients (98%) required no more than 7 days of intramuscular clozapine treatment before transitioning to the oral product. And 90% of patients remained compliant with subsequent oral clozapine treatment.

In a study by Schulte and colleagues [34], 6 out of 10 patients who received at least one clozapine injection successfully transitioned to the oral product. The remaining patients had to stop intramuscular clozapine either due to side effects but also due to long-term exposure not leading to successful transition (more than 30 days in 3 patients). In those patients who were successfully transitioned to oral clozapine, improvement in their condition was sustained during the long-term follow-up for a mean of 15.2 months.

In a study by Holmes [21], all 7 patients receiving intramuscular clozapine were successfully transitioned to oral clozapine after no more than 4 injections. All patients have improved at least somewhat.

In a case series by Henry and colleagues [23], 6 out of 8 patients treated with intramuscular clozapine successfully transitioned to the oral product. The majority of them was showing clinical improvement by the end of 5 months of the observation period.

In a retrospective study by Casetta and colleagues [22], 16 out of 19 patients receiving intramuscular clozapine were successfully transitioned to the oral formulation. The authors also reported on the long-term effects of enforced clozapine and found out that clozapine discontinuation rates at 2-year follow-up were similar to a comparison group of patients who were prescribed only oral clozapine.

In summary, there is clear evidence in all published studies suggesting that more than half of patients treated with intramuscular clozapine may successfully transition to the oral formulation and that most of them need only a few parenteral injections.

Adverse effects and safety considerations

Most adverse effects associated with intramuscular administration of clozapine are similar in their presentation and frequency to adverse effects reported after its oral administration [38], but it has to be noted that none of the reported studies were powered or designed to evaluate safety.

Reported adverse effects include agranulocytosis and neutropenia [22, 32, 34], constipation [4], hypersalivation [4, 34], pneumonia [22], and impaired liver function [33].

However, several adverse effects common with clozapine may have a different intensity and time-course after intramuscular administration due to faster systemic absorption and transiently elevated blood levels that may influence its safety. Those include sedation, cardiovascular adverse effects, and possible seizure activity. 

Sedation appears to be more pronounced and with faster onset after intramuscular administration, but that side effect was considered to be beneficial [4]. A study investigating possible beneficial effects of intramuscular clozapine in aggressive schizophrenic patients with agitation has been even proposed, but no outcomes were reported [39].

Tachycardia and hypotension following clozapine administration also have faster onset after the intramuscular delivery route and are seen with lower clozapine doses [31]. Of note, cardiovascular arrest finally leading to death has been reported after intramuscular clozapine administration, but it was given in combination with haloperidol in a patient with a history of a septal defect [30]. However, intramuscular clozapine has been safely administered to a patient with heart failure [24].

Even though seizure activity induced by clozapine seems to be related to its blood levels, there are no published reports of seizures following its intramuscular administration. However, in one case report, a patient developed dyskinesia-like side effects immediately after administration of 100 mg of intramuscular clozapine [35], and initiation of seizures often presents like that [38]. Caution in patients with pre-existent seizures is definitely needed.

Interestingly, knowledge of possible safety risks associated with intramuscular clozapine, especially in the clozapine-naïve population, reached even the movie industry as intramuscular clozapine was implemented as a fictional murder weapon in the highly popular television series The Walking Dead. In one of its episodes, the actress grinded up clozapine tablets with a mortar and prepared an injection from the powder. She then proceeded to inject the fictional patient intramuscularly. The treated patient developed tonic-clonic seizures and, in a few minutes, he died [40]. 

In addition to systemic adverse effects, intramuscular clozapine injections are reported to be painful, and injection site swelling and formation of nodules have been reported. From available evidence, it appears that nodules are more common with a long-term administration of higher clozapine doses [22-23].

Discussion and future directions

Though intramuscular clozapine has been reported only in generally small studies, it has been demonstrated that its use may facilitate successful transition to oral clozapine in most patients, and it definitely represents a valuable tool for addressing refusal of oral clozapine in treatment-refractory patients as well as administration of clozapine to patients who are unable to accept oral formulations. However, it has to be noted that all published studies were retrospective case series only and it is thus difficult to draw any broader or evidence-based conclusions from them. Most importantly, evidence for long-term benefits of this approach remains limited. The nature of the indication (back-up injection for refusal of oral medication), however, makes any design of better controlled randomized or blinded studies not feasible.

In spite of clinical benefits of intramuscular clozapine, several issues need to be addressed before its broader use could be implemented. Current formulations of clozapine are unlicensed products, and their safety was thus not evaluated and vetted by regulatory authorities [20]. That factor is important as most patients are given intramuscular clozapine against their will, and without being completely sure that no harm is done, the use of the product needs to be limited to patients where no other options are available as enforcing of clozapine has significant ethical implications [41-42].

Though available evidence suggests that it is generally well-tolerated, our knowledge of its safety after rapid administration still remains limited as there are nuanced differences between cardiovascular and sedative effects of oral and intramuscular formulations. It would be really helpful if more in-depth and thorough cardiovascular safety and pharmacokinetic studies of intramuscular clozapine were conducted. Without having those available, it is advisable to screen the patients appropriately prior to intramuscular clozapine administration and to monitor their vital signs carefully based on established protocols [26]. 

Rapid administration of clozapine may also lead to a higher risk of drug-drug interactions, and unless more combination studies become available, it would be wise not to co-administer intramuscular clozapine with other intramuscular agents, mainly other antipsychotics and benzodiazepines.

One of the unique side effects of intramuscular clozapine is the formation of local nodules. Additional formulation work increasing local tolerability of clozapine solution and possibly also making a higher-strength solution for injection [23] would also be very helpful.

Let’s hope that the intramuscular clozapine product attracts more attention from drug-delivery and pharmaceutical industries which could lead to more studies and possible regulatory approval for this product in the future. That would make its broader usage more feasible.

For now, we encourage our colleagues who have access to intramuscular clozapine to report on their experience, especially regarding adverse effects, so that we would gain more reassurance on its safe use in the vulnerable population of patients with treatment-refractory schizophrenia.

## Conclusions

Overall, intramuscular clozapine can present a convenient alternative for patients with treatment-refractory schizophrenia who refuse or cannot take oral clozapine treatment. Multiple generally small studies have suggested that a majority of patients might successfully transition to oral clozapine within a few days of intramuscular treatment. However, it has to be noted that all published studies were only retrospective case series and it is thus difficult to draw any broader or evidence-based conclusions from them. Given these results and clozapine’s documented side-effect profile, more studies, mainly on its safety, are needed to better understand this approach’s capabilities and limitations.
